# Effects of Dietary Carbohydrate Profile on Nocturnal Metabolism, Sleep, and Wellbeing: A Review

**DOI:** 10.3389/fpubh.2022.931781

**Published:** 2022-07-13

**Authors:** Konstantinos Mantantzis, Vanessa Campos, Christian Darimont, Francois-Pierre Martin

**Affiliations:** Nestlé Institute of Health Sciences, Nestlé Research, Société des Produits Nestlé S.A., Lausanne, Switzerland

**Keywords:** carbohydrates, fiber, nocturnal metabolism, protein, sleep quality, wellbeing

## Abstract

Sleep is a crucial biological function and a well-established driver of health and wellbeing across the lifespan. In this review, we describe how sleep in humans is associated with specific circadian metabolic and physiological changes, and how the organization of sleep-wake states is related to regulation of nocturnal metabolism during fasting. Among the modifiable factors that can contribute to sleep-related benefits, emerging evidence suggests that diet and nocturnal changes in glucose regulation are strong determinants of sleep quality. Here, we review studies that have explored the importance of quantity and quality of dietary carbohydrates and proteins in modulation of sleep and sleep-related health benefits. Future research may guide the creation of nutritional solutions to improve sleep, which could lead to positive changes in health, wellbeing, and overall quality of life.

## Introduction: Importance of Sleep for Wellbeing

Good sleep quality has been associated with a wide range of benefits, including optimal cognitive function, mood and mental performance, better cardio-metabolic health and immunity (1). Conversely, poor sleep both in terms of duration and overall quality has been linked to a host of negative consequences for overall health and wellbeing, including behavioral problems, increased risk of metabolic syndrome and cancer (2), as well as increased risk of mortality across different age groups (3).

Quality of sleep has been associated with wellbeing and behavioral benefits across the lifespan (4–6). The majority of evidence investigating the role of sleep for next-day behavior comes from sleep deprivation studies, clearly showing that both sleep disruption and deprivation can negatively impact aspects of cognition, including declarative memory, memory encoding and recall, as well as cognitive flexibility (6). Among the cognitive domains strongly influenced by sleep, levels of daytime vigilance and subjective alertness are strongly related with sleep duration (7). In fact, the link between cognition and sleep quality is so prevalent that performance on vigilance tasks in which participants have to remain focused on a specific activity for extended periods of time has been used as a measure of sleep loss (8). Sleep loss is also considered a risk factor for psychological wellbeing, a link that has been partly attributed to sleep loss-related disturbances in emotion regulation capacity (4). For example, studies have found that sleep deprivation can cause an increase of almost 60% in amygdala activity (9), as well as an increase in pupil diameter (10) when subjects are presented with negative stimuli, suggesting that poor sleep can lead to increased emotional reactivity to negative information.

As such, interventions to improve sleep quality could lead to significant improvements in quality of life and psychological wellbeing (11). Among the modifiable factors that can influence sleep quality, there is now emerging evidence suggesting that our diet and its impact on nocturnal changes in the metabolic regulation of hormone release can affect sleep parameters (12, 13).

### Sleep and Cardiometabolic Health

Recent developments in our understanding of the relation between circadian rhythmicity and human health have highlighted the importance of sleep for metabolic health. The link between sleep quality and metabolic disease risk has received a lot of attention, and has provided evidence of a bidirectional association between sleep disruptions, obesity, and disturbances in glucose metabolism (14). Disturbed sleep alters metabolic pathways and contributes to insulin resistance, as well as deregulation of energy expenditure and appetite, contributing to an increased disease risk. For instance, both short and longer-than-normal sleep duration have been associated with greater risk for development of type 2 diabetes and weight gain (15, 16). It has been hypothesized that the link between disturbed sleep and metabolic changes could be mediated by perturbations in eating behaviors, including increased energy intake, as well as higher frequency and number of meals and snacks consumed per day (17). Furthermore, psychological and metabolic deregulations occurring in individuals with obesity and diabetes further interfere with poor sleep, reinforcing a negative health impact and creating a virtuous cycle.

An example of the link between sleep and cardiometabolic health comes from shift workers, which in Europe includes those working outside of regular office hours (e.g., 09:00–17:00), as well as night shifts where work routinely takes place between 22:00 and 05:00. Shift work has been associated with a disruption of circadian rhythm and normal sleep patterns, a condition commonly referred to as shift work disorder (18). In line with evidence showing a link between sleep and cardiometabolic health, a recent review has suggested that shift work poses a risk factor for cardiovascular risk, obesity, diabetes and metabolic syndrome, and can lead to changes in eating behaviors (irregular meal patterns and timing) and higher propensity for unhealthy food choices (19).

Melatonin is a central regulator of sleep and circadian metabolism, with known acute and circadian phase-shifting effects, including possible impact on glucose control (20, 21). In humans, the peak in melatonin during nighttime coincides with a reduction in glucose tolerance (20). Garaulet et al. recently discussed that eating meals (high glycemic meals, in particular) close to the moment of exogenous melatonin intake or during the night when endogenous melatonin levels are high, can have deleterious effects on glycemic control (20). They proposed a so-called “timing model,” in which they describe that low melatonin during eating periods supports high glucose tolerance, while high melatonin during fasting could facilitate β-cell recovery (20). Therefore, the unphysiological state of high circulating melatonin levels concurrent with food intake, as seen in nocturnally feeding populations, shift-workers or users of exogenous melatonin, may result in dysregulation of glucose metabolism and contribute to an increased risk of T2D development (20).

## Sleep and Nocturnal Metabolism

Sleep is a natural state of rest characterized by reduced or absent consciousness, reduced sensory activity and voluntary muscle inactivity (22). Typical sleep architecture is comprised of two components: non-rapid eye movement (NREM) and rapid eye movement (REM) sleep. Overall, sleep quality is thought to be driven by the total duration of slow wave sleep (SWS), which constitutes the third (and deepest) phase of NREM (23, 24).

SWS and REM are associated with distinct physiological states, including variations in respiration, muscle tone, heart rate, blood pressure and flow to tissues and organs, body temperature, nocturnal energy (e.g., energy store restoration and substrate oxidation) and endocrine metabolism (22, 25). Considering that sleep in humans is generally consolidated in a single 7- to 9-h period, an important metabolic consequence of this is that an extended period of total fasting state must be maintained overnight (25). Numerous studies have shown that despite the prolonged overnight fast, glucose levels remain relatively stable or decrease minimally when compared to daytime fasting. For instance, daytime fasting results in a decrease of blood glucose from 5.3 to 4.2 mmol/L in healthy subjects, whereas nocturnal glycemia oscillates around 5.0 mmol/L (25). Overnight, the blood glucose variations display different patterns. First, nocturnal sleep is marked by a decrease in hepatic glucose output which could be partially regulated by peripheral signals derived from lipolysis (e.g., decreased plasma glycerol and free fatty acids) (26, 27). Studies employing glucose infusion methodologies have shown a concurrent reduction in glucose utilization during sleep (14, 25). To maintain glucose level relatively stable during nocturnal sleep, a number of mechanisms intervene, including the action of counterregulatory hormones to modulate glucose production and utilization (25). For example, during the first hours of sleep there is a decrease in cortisol and epinephrine concentrations, coupled by an increase in growth hormone secretion which is posited to promote glucose stability through the inhibition of glucose uptake by muscle (28). Following this stage, secretion of growth hormone is inhibited while epinephrine and cortisol concentrations tend to increase, eventually reaching daytime level. In several overnight studies, a rise in blood glucose and insulin levels has been observed toward the end of the nocturnal sleep period. This is known as the dawn phenomenon and is primarily observed among diabetic patients and less often in healthy populations (25).

Furthermore, sleep studies have uncovered a close relationship between brain glucose metabolism, blood glucose and sleep stages. In particular, increases in plasma glucose appear to partially reflect the predominance of REM stages in early sleep, while brain glucose utilization is reduced during the NREM stage and contributes to a two-third fall in glucose utilization during sleep (14, 25).

### Associations Between Glycemic Traits and Sleep Parameters

Numerous studies have investigated the link between glycemic traits and sleep quality. The emerging evidence suggests a bidirectional association between sleep and metabolic parameters, with sleep both affecting and being affected by glycemic control and metabolic status [e.g., diabetes (29)].

Firstly, diabetics have poorer sleep patterns than healthy individuals, including more subjective sleep complaints, lower sleep duration, and higher incidence of sleep apnea (30). Secondly, poor glucose control is associated with lower sleep quality among those diagnosed with diabetes and pre-diabetes (31–33). For example, poor sleepers have been found to exhibit ~23% higher fasting blood glucose and 48% higher fasting blood insulin levels than individuals with typical sleep patterns (31–33). Sleep characteristics were found to be associated to HbA1c plasma levels, which may indicate that control of both fasting glucose and the glucose response to a meal could be linked to sleep regulation (32). Indeed, different sleep parameters (e.g., duration, variability and subjective complaints) have been found to be associated with glucose management status. A few studies have reported associations between glucose tolerance and sleep parameters under controlled dietary or therapeutic glucose management conditions, with evidence of improved glucose control being linked to improvements in sleep quality (34, 35).

Beyond the potential effect of glycemia on sleep, sleep problems including sleep apnea, insomnia and even subjective complaints of daytime sleepiness have been linked to higher insulin resistance and glucose intolerance in healthy subjects (36). Among the different sleep stages, experimental suppression of SWS (one of the key drivers of sleep quality), but not REM, has been linked with increased morning glucose and insulin responses, as well as reduced postprandial insulin sensitivity in healthy individuals (37). In healthy young and middle-aged adults with insomnia, physiologic hyperarousal has been associated with nocturnal insulin secretion and insulin resistance, as well as postabsorptive carbohydrate availability and increased fuel needs of glucose-dependent and glucose-independent tissues (38, 39).

### The Role of Dietary Carbohydrates and Proteins in Sleep

Reviews on the complex interactions between diet and sleep have pinpointed the role of both macro and micronutrients (13). Specifically, there is evidence that the macronutrient composition of the diet, and especially evening meals, can significantly affect nocturnal metabolism and sleep quality (13). Overconsumption of a high-fructose hypercaloric diet has been reported to result in higher sleeping metabolic rate and increased urinary excretion of cortisol compared to a similar overfeeding diet in which carbohydrates come from whole-wheat foods (40). Such clinical evidence may be indicative of the role of dietary carbohydrate profile in promoting different hormonal responses to modulate nocturnal glucose metabolism. Further work has focused on the importance of carbohydrate intake for sleep quality, with increasing evidence pointing toward the importance of carbohydrate quality in particular as a determinant of sleep quality (41–47). Specifically, higher carbohydrate quality, e.g., diets with low glycemic index and rich in fibers, have been linked to lower risk of insomnia and better sleep quality (48–50). Conversely, diets with a high glycemic index, or diets rich in added sugars, starch and refined grains have been associated with higher prevalence of sleep complaints (48, 49).

Other studies have captured the importance of protein intake in the evening for sleep (51, 52). In fact, individuals characterized as good sleepers (e.g., sleep duration >7 h, global sleep score <5, sleep latency <30 min, and sleep efficiency >85%) have been found to have a higher energy distribution from dietary protein and a lower percentage of energy from dietary carbohydrate and fat compared to poor sleepers (51, 52). The evidence on the beneficial role of protein has been supported by further studies suggesting that diets rich in protein (e.g., 20–30% of meal energy) can lead to a decrease in the number of awakenings during the night, therefore minimizing sleep fragmentation and improving sleep quality (42, 51).

The effects of diet on sleep are mediated by numerous variables, including meal timing (13, 53). For example, Gu et al. (54) assessed the effects of late vs. routine time of dinner (i.e., 22:00 vs. 18:00) on nocturnal metabolism and found that a dinner consumed closer to bedtime can lead to glucose intolerance during sleep. Pizinger et al. (55) further explored how meal and sleep timing interact to influence overnight glucose and insulin levels/release and have concluded that delays in bedtime coupled with eating late in the day could contribute to increased risk of diabetes and other negative health outcomes. More recently, Chung et al. (56) examined the role of meal timing for sleep quality. The authors found that meal intake within 3 h before bedtime can lead to more awakenings during the night which, in turn, can influence overall sleep quality due to sleep fragmentation.

### Metabolic Response to Carbohydrate Intake and Sleep

Results of interventional studies have further consolidated the importance of carbohydrate composition of the evening meal on sleep through the modulation of nocturnal glucose and carbohydrate oxidation. In particular, increased nocturnal carbohydrate oxidation can suppress SWS, in line with findings of reciprocal changes in REM and SWS following manipulation of carbohydrate quality and quantity of a meal (49). Consumption of high-carbohydrate meals shortly before sleep has been associated with higher nocturnal blood glucose levels and a reduction in SWS (57), with the negative effects extending beyond the duration of the intervention. However, carbohydrate-based high-glycemic index meals consumed 4 h before sleep have been found to support easier transition into sleep (shorter sleep onset latency) (43), further underlying the complicated nature of the interactions between meal timing and composition.

The mechanisms of action behind the role of carbohydrates for sleep remain poorly understood. One mode of action could relate to the fact that high glycemic response to evening meals may result in perturbation of nocturnal carbohydrate metabolism and decreased sleep quality (48). Postprandial hyperglycemia caused by a high dietary glycemic load and the resultant compensatory hyperinsulinemia can lower plasma glucose to concentrations that could compromise brain glucose (3.8 mg/dL), triggering the secretion of autonomic counterregulatory hormones such as adrenaline, cortisol, glucagon, and growth hormone (48). Symptoms of counter-regulatory hormone responses include heart palpitations, tremor, cold sweats, anxiety, irritability, and hunger, which could all be plausibly linked to reduced sleep quality. In addition, hypoglycemic events have been shown to cause awakenings during sleep, which is known to compromise sleep efficiency even in healthy adults (58).

### Protein-Carbohydrate Interactions and Importance of Tryptophan Metabolism

Macronutrients can also affect sleep via their effects on other circulating bioactives or micronutrients. For example, dietary protein and carbohydrate intake could influence factors relevant for the synthesis of serotonin and melatonin which, in turn, have been linked with sleep quality (13, 59, 60). Synthesis of serotonin and melatonin is influenced by the availability of tryptophan, an amino acid that can promote relaxation and facilitate sleep initiation. The macronutrient composition of evening meals, and particularly the carbohydrate-to-protein ratio, has been found to increase the ratio of tryptophan-to-other large neutral amino acids (61), which is believed to facilitate tryptophan's capacity to cross the blood-brain barrier and boost serotonin and melatonin synthesis (62). In turn, this could facilitate sleep onset. With regards to the mechanism of action, meals high in carbohydrates can promote higher tryptophan-to-large neutral amino acid ratio by stimulating the uptake of competing amino acids into muscle, thereby allowing tryptophan to be more available to cross the blood-brain barrier (48, 60). This phenomenon could explain findings of a positive influence of high-carbohydrate meal intake 4 h before bedtime on sleep initiation (43). However, despite high-carbohydrate meals potentially promoting easier transition to sleep via purported increases in tryptophan brain availability, the compensatory hyperinsulinemia and counterregulatory hormonal responses following the consumer of a high-carbohydrate meal can cause sleep fragmentation and decrease sleep quality throughout the night (48).

### Nocturnal Glycemia and Next-Day Behavioral Benefits

The association between nocturnal glycemia and next-day benefits related to cognition, mood and wellbeing is less well-understood and available evidence is scarce. With regards to cognition, there is a well-established link between sleep quality and cognitive performance. Among the different sleep stages, it has been proposed that SWS is more closely linked to declarative memory, whereas REM sleep underlies the ability to synthesize abstract information such as detecting patterns in newly acquired information (non-declarative) (5, 6). More recent views on the influence of the different sleep stages for cognitive performance have suggested that SWS and REM might have complementary roles in the consolidation of newly acquired information [for different theories, see (5)]. Experimentally-induced nocturnal hypoglycemia during SWS, achieved through insulin infusion to stabilize blood glucose levels to 2.2 mmol/L, has been associated with worse memory the next day (63).

In a similar manner, studies manipulating blood glucose levels to remain within the range of 2.3–2.7 mmol/L during SWS have uncovered lower levels of wellbeing, measured as a decrease in self-reported vitality and feelings of contentment (64). It should be noted that most studies linking nocturnal glycemia with cognitive and mood benefits have been performed in diabetics. Therefore, there is a significant gap in our understanding of how nocturnal glycemia is associated with next-day benefits in healthy populations and would need to be addressed in future studies.

To date, no studies have investigated the role of evening meal composition for next-day benefits via sleep improvement as one of the mechanisms through which it might affect subjective and objective cognitive performance and mood. A study on the carbohydrate content of an evening meal and next-day breakfast has shown that high GI meals in the evening can improve cognitive performance the next day when coupled with a high GI breakfast (65). It should be noted that this study did not include measures of sleep, so the role of sleep quality as a mediating factor cannot be determined. The lack of further evidence necessitates more studies into the role of evening meals for behavioral outcomes.

## Conclusions and Perspectives

There is a recognized link between carbohydrate metabolism, meal timing and sleep health. Current research shows that the composition and timing of the evening meal can have profound effects of sleep. Despite established circadian mechanisms of glucose metabolism and distinct nocturnal changes in metabolism, the mode of action of dietary carbohydrates remains poorly understood, and its elucidation will require novel circadian research. Such findings could guide the creation of nutritional solutions to improve sleep and sleep-related benefits.

## Author Contributions

F-PM and KM conceived and performed the review. F-PM, KM, VC, and CD drafted the manuscript. All authors read and approved the final version of the manuscript.

## Conflict of Interest

VC, and CD are employees of Société des Produits Nestlé S.A., Switzerland. F-PM was an employee of Société des Produits Nestlé S.A., Switzerland, at the time of the review and is currently an employee at H&H Group, Switzerland. KM was an employee of Société des Produits Nestlé S.A., Switzerland, at the time of the review and is currently an employee at GSK, Switzerland.

## Publisher's Note

All claims expressed in this article are solely those of the authors and do not necessarily represent those of their affiliated organizations, or those of the publisher, the editors and the reviewers. Any product that may be evaluated in this article, or claim that may be made by its manufacturer, is not guaranteed or endorsed by the publisher.
